# Cloning, expression and antiviral activity of mink alpha-interferons

**DOI:** 10.1186/s12917-015-0359-z

**Published:** 2015-02-21

**Authors:** Hai-ling Zhang, Jian-jun Zhao, Xiu-li Chai, Lei Zhang, Xue Bai, Bo Hu, Hao Liu, Dong-liang Zhang, Ming Ye, Wei Wu, Xi-jun Yan

**Affiliations:** Division of Infectious Diseases of Special Economic Animal, Institute of Special Animal and Plant Sciences, Chinese Academy of Agricultural Sciences, 4899 Juye Street, Changchun, 130112 China; Jilin Teyan Biotechnological Co. Ltd, 388 Liuying West Road, Changchun, 130122 China

**Keywords:** Mink, Interferon subtype, Antiviral activity

## Abstract

**Background:**

As a key link between innate and adaptive immune responses, the interferon (IFN) system is the first line of defense against viral infection. IFN, and in particular, IFN-α, has been used clinically as an effective therapeutic agent for viral infections. However, different subtypes of IFN-α demonstrate distinct antiviral activity. Therefore, it is important to identify IFN-α subtypes with high antiviral activity for the development of genetically engineered antiviral drugs.

**Results:**

In this study, we cloned the genes for 13 IFN-α subtypes from peripheral blood lymphocytes of the mink. The homologies of the 13 mink IFN-α genes were 93.6–99.3% and 88.8–98.4% at the nucleotide and amino acid sequence levels, respectively. In contrast to human and canine IFN-α subtypes, most mink IFN-α subtypes contained two N-glycosylation sites. We expressed and purified 13 mink IFN-α subtypes in *Escherichia coli*. The cytopathic effect inhibition assay showed that all the 13 recombinant mink IFN-α subtypes inhibited the propagation of vesicular stomatitis virus in WISH cells, with IFN-α2 and IFN-α12 demonstrating the highest activities. Furthermore, recombinant mink IFN-α2 and IFN-α12 significantly suppressed the propagation of canine distemper virus in Vero cells, with IFN-α2 demonstrating the highest activity.

**Conclusions:**

We identified the mink IFN-α2 subtype as a promising candidate for the development of effective antiviral drugs.

## Background

Interferon (IFN) was first identified by Isaacs in 1957 [1]. IFN belongs to the cytokine family of proteins and has a wide range of physiological functions, such as inhibiting viral infection, regulating cell proliferation and differentiation, and modulating immune responses [2]. The IFN system is one of the first defensive barriers against viral infection and is an important component of natural antiviral immunity [3,4].

IFNs are key cytokines with antiviral, antitumor, and immunomodulatory activities. Based on gene sequences, chromosome location, and receptor specificity, the members of the IFN family are classified into I, II, and III subtypes. Subtype I includes IFN-α, β, ω, ε, κ, δ and ζ [5-8], and type I IFNs have been shown to possess effective antiviral activity. Type II IFN is also known as immune IFN (IFN-γ) [9]. Interferon-γ, a cytokine produced by T lymphocytes and natural killer cells, plays a central role in the modulation of the immune response [10]. The type III interferons are also known as the IFN-λs and are more related to type I IFNs based on their amino acid sequence and protein function. However, the more limited tissue expression of IFN-λ receptors suggests that type III IFNs do not simply recapitulate the type I IFN antiviral system. They have antiviral effects in the respiratory tract, gastrointestinal tract, skin mucosa, epithelial cells, and some tumor cells. Among the IFN family, IFN-α is one of the major modulators of the defensive system against viral infection in mammals and has been widely used in the clinic as a therapeutic for viral infection [11,12]. All IFNs identified in vertebrates are secreted proteins, with IFN-α being secreted from virus-infected white blood cells. The human IFN family consists of 13 subtypes. Despite differences in their amino acid sequences, the subtypes of human IFN-α have similar 3D structures and similar physiological and biochemical characteristics, such as stability and recognition by surface receptors. Human IFN-α is composed of 166–172 amino acids and has no glycosylation sites. The average molecular weight of IFN-α is about 19 kDa. The homology of amino acids among IFN-αs from different subtypes ranges from about 75–95% [13]. The IFN-αs can be further classified into subtypes, which have different biological functions.

IFN is a conserved molecule that has been found in humans, mouse, sheep, rabbits, dogs, weasels and other mammals, as well as in fish, turtles and insects. IFNs have been used in both human and veterinary medicine, not only for the treatment of viral infections but also for cancer. Mink (*Neovison vison*) is one of the major fur animals and its health problems have become an important issue for the fur industry. Infection by the canine distemper virus, Parvovirus, and Aleutian mink disease virus among minks has caused serious economic losses to the fur animal industry [14]. Therefore, there is an urgent need to develop effective, non-toxic, and environmentally friendly antiviral drugs to control viral infections in mink. In this study, we cloned and expressed the genes for 13 IFN-α subtypes in the mink and characterized their antiviral activity.

## Results

### Analysis of mink IFN-α genes

By reverse transcription polymerase chain reaction (RT-PCR), we successfully amplified the predicted 564 bp mink IFN-α (MiIFN-α) cDNA. Restriction enzyme analysis confirmed that we had correctly subcloned MiIFN-α cDNAs into recombinant plasmids. DNAStar software analysis demonstrated that the cDNAs of 13 subtypes of MiIFN-α were 564 bp long and encoded 187 amino acids. Due to the different amino acids components, there were slight variations in molecular weight among the 13 MiIFN-α subtypes. The GenBank registration number and molecular weight of the cloned 13 MiIFN-α subtypes are listed in Table 1.

**Table 1:** **The 13 MiIFN-α subtypes cloned in this study**

**IFN-α subtype**	**MW (Da)**	**Accession number**
MiIFN-α1	20956	EU863613
MiIFN-α2	20999	EU863614
MiIFN-α3	20958	EU863615
MiIFN-α4	21031	EU863616
MiIFN-α5	21089	EU863617
MiIFN-α6	21204	EU863618
MiIFN-α7	21002	EU863619
MiIFN-α8	21032	EU863620
MiIFN-α9	21021	EU863621
MiIFN-α10	21146	EU863622
MiIFN-α11	21248	EU863623
MiIFN-α12	21060	EU863624
IFN-α13	20983	EU091340

SignalP 4.1 server software (http://www.CBS.DTU.DK/Services/SignalP) analysis predicted that the N terminal 23 amino acids represented the signal peptide. The homologies of 13 MiIFN-α subtypes were 93.6–99.3% and 88.8–98.3% at the nucleotide and amino acid sequence levels, respectively (Table 2).

**Table 2:** **Homology (%) of amino acids and nucleotides among MiIFN-α subtypes**

	**IFN-α1**	**IFN-α2**	**IFN-α3**	**IFN-α4**	**IFN-α5**	**IFN-α6**	**IFN-α7**	**IFN-α8**	**IFN-α9**	**IFN-α10**	**IFN-α11**	**IFN-α12**	**IFN-α13**
IFN-α1	-	99.3	97.0	95.0	96.3	94.5	94.7	94.7	93.8	94.7	94.3	96.1	95.4
IFN-α2	98.4	-	96.6	95.7	97.0	95.2	94.7	94.9	94.0	94.3	94.0	95.7	95.0
IFN-α3	94.7	94.1	-	96.3	96.8	94.7	96.3	96.6	95.7	96.3	95.2	98.0	95.6
IFN-α4	90.4	92.0	93.0	-	98.8	98.4	96.5	96.3	95.4	96.6	95.0	95.7	96.1
IFN-α5	93.0	94.7	93.6	97.3	-	97.2	97.0	96.1	95.2	97.0	95.6	95.6	96.1
IFN-α6	89.3	90.9	91.4	98.4	95.7	-	95.2	95.0	94.3	95.0	96.3	94.7	95.6
IFN-α7	91.4	90.9	93.0	93.6	94.1	92.5	-	96.8	96.3	97.9	97.2	95.2	96.3
IFN-α8	90.9	90.9	94.1	92.5	92.0	91.4	94.1	-	99.1	95.9	94.3	97.3	95.6
IFN-α9	89.3	89.3	92.5	90.9	90.4	89.8	93.6	98.4	-	95.0	93.6	96.5	94.7
IFN-α10	91.4	90.9	94.1	94.7	95.2	93.0	96.8	92.0	90.4	-	97.2	96.3	96.3
IFN-α11	89.8	89.3	92.0	92.5	93.0	94.1	95.2	90.4	88.8	96.8	-	94.9	95.7
IFN-α12	93.0	92.5	97.3	92.5	92.0	91.4	92.0	95.2	93.6	93.6	92.0	-	95.6
IFN-α13	91.4	90.9	91.4	92.0	92.5	91.4	93.6	90.4	88.8	93.6	93.0	90.4	-

“–” same sequence. The upper line shows identities at the nucleotide level; The lower line shows identities at the amino acid level. The sequences for alignment were from the following GenBank accession numbers: IFN-α1(MiIFN-α1, EU863613), IFN-α2 (MiIFN-α2, EU863614), IFN-α3 (MiIFN-α3, EU863615), IFN-α4 (MiIFN-α4, EU863616), IFN-α5 (MiIFN-α5, EU863617), IFN-α6 (MiIFN-α6, EU863618), IFN-α7(MiIFN-α7, EU863619), IFN-α8 (MiIFN-α8, EU863620), IFN-α9 (MiIFN-α9, EU863621), IFN-α10 (MiIFN-α10, EU863622), IFN-α11 (MiIFN-α11, EU863623), IFN-α12 (MiIFN-α12, EU863624), IFN-α13 (MiIFN-α13, EU091340).

**Figure 1:**
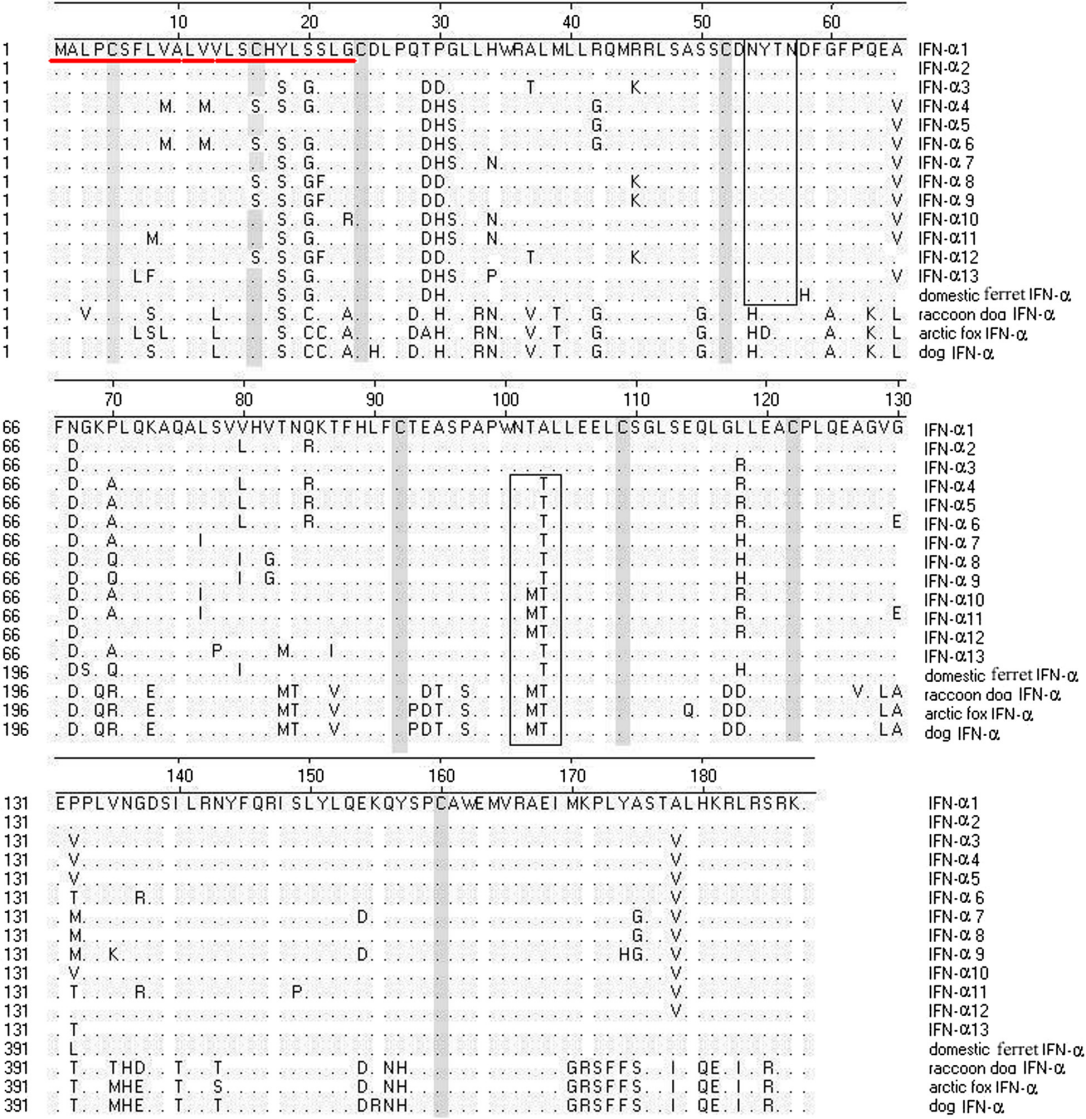
**Analysis of amino acid sequences of 13 MiIFN-α subtypes.** Red underlining indicates the predicted signal sequence of MiIFN-αs. Shaded areas represent the predicted cysteine residues, and boxed areas indicate potential N-glycosylation sites.

Most secreted proteins in eukaryotes are modified on the amino acid Asn located in the consensus sequence Asn.Xaa.Ser/Thr (NXS/T) by an N-glycan, a process known as N-glycosylation [15]. Analysis using NetNGlyc Server 1.0 (http://www.CBS.DTU.DK/Services/NetNGlyc) predicted that IFN-α1–3 contained one glycosylation site at ^54^NYTN^57^, whereas IFN-α4–13 contained two glycosylation sites. In addition to the glycosylation site at ^54^NYTN^57^, IFN-α4–9 and IFN-α13 contained a glycosylation site at ^101^NTTL^104^, whereas IFN-α10–12 contained a glycosylation site at ^101^NMTL^104^. Further analysis demonstrated that the number and position of cysteines were not the same among the 13 subtypes. IFN-α1–3, IFN-α5, IFN-α7, IFN-α10, IFN-α11 and IFN-α13 contained 8 cysteine residues at the same positions of 5, 16, 24, 52, 92, 109, 122 and 160. IFN-α4, IFN-α6, IFN-α8, IFN-α9 and IFN-α12 contained only 7 cysteine residues with a cysteine residue at position 16 replaced by serine (Figure 1). These data demonstrate the existence of a single nucleotide polymorphism (SNP) among MiIFN-α subtypes, in agreement with previous reports on SNPs of IFN-α in other species [15,16].

**Figure 2:**
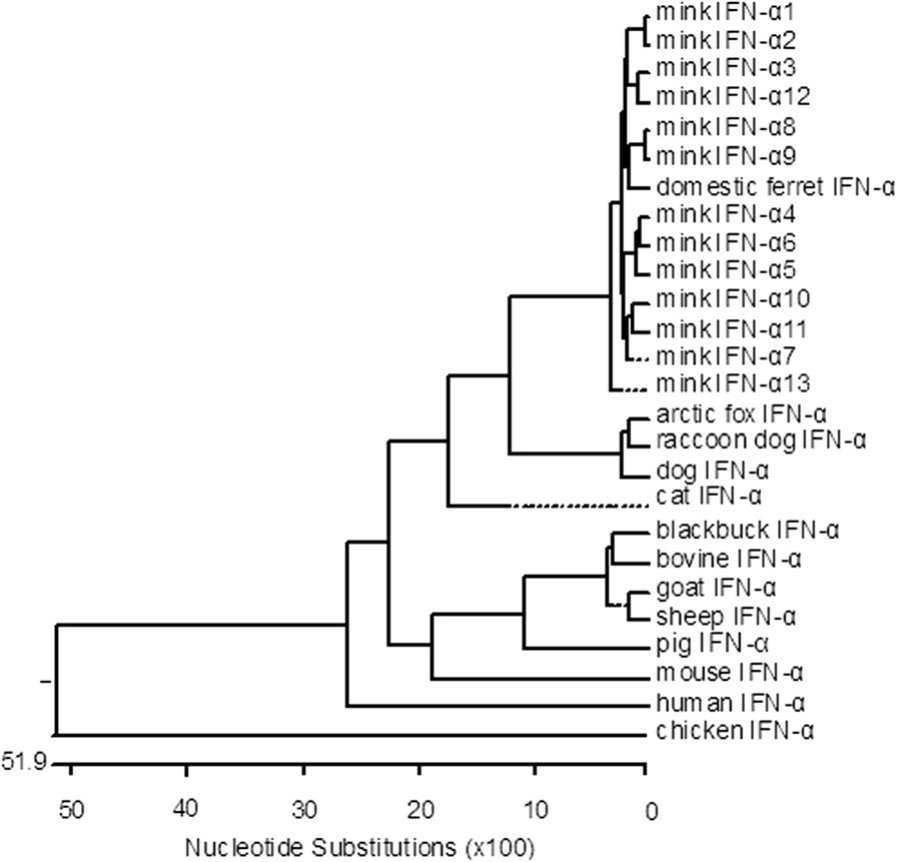
**Phylogenetic tree of IFN-α nucleotide sequences of several animal species.**

Both mink and ferret belong to *Mustelidae*. The homology between IFN-α1–13 and the published sequences for ferret (GenBank registration number: EF368207) were 95.0–97.3% and 91.5–94.2% at the nucleotide and amino acid sequence levels, respectively. The amino acid sequence analysis showed that fox (EF990625), raccoon (EF543192), and canine (EF28625) IFN-α each contained only one potential N-glycosylation site at ^101^NMTL^104^, which is the same site in MiIFN-α10–12. The homologies between mink, fox, raccoon and canine IFN-α were 79.1–80.9% and 68.8–71.4% at the nucleotide and amino acid sequence levels, respectively. Nucleotide evolution tree analysis showed significant differences in the nucleotide sequences among the 13 subtypes of MiIFN-α and those of other species of animals and human IFN-αs (Figure 2).

### Expression and purification of MiIFN-α in *E. coli*

**Figure 3:**
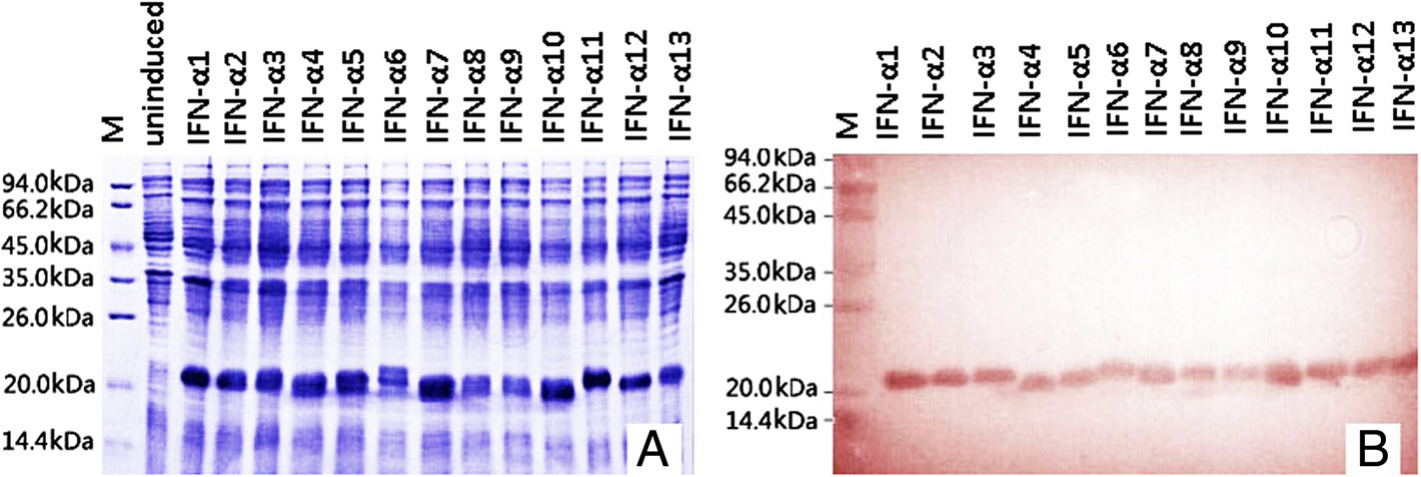
**SDS-PAGE analysis of recombinant MiIFN**-**αs expressed in** ***E. coli*** **induced by IPTG (A).** Western blot analysis of His-tag recombinant MiIFN-αs by 6-poly histidine monoclonal antibodies **(B)**.

To evaluate the biological activity of MiIFN-α, we subcloned 13 MiIFN-α cDNAs into a prokaryotic expression vector pProEX HTb and induced their expression by isopropyl-beta-D-thiogalactopyranoside (IPTG) in *E. coli* BL21. Sodium dodecyl sulfate (SDS)-polyacrylamide gel electrophoresis (PAGE) analysis showed the presence of protein bands at 19 kDa (Figure 3A), consistent with the predicted molecular weights of MiIFN-α. Protein solubility analysis showed that MiIFN-α recombinant proteins were in the form of inclusion bodies. SDS-PAGE analysis of the isolated inclusion bodies showed that 80% of recombinant proteins were in inclusion bodies. Western blot analysis of partially purified MiIFN-α with an anti-6xHis antibody showed a specific band of about 19 kDa (Figure 3B).

### Antiviral activity of recombinant MiIFN-α

To examine the antiviral activity of recombinant MiIFN-αs, we first used the WISH/ vesicular stomatitis virus (VSV) system. Compared to the cells without virus infection, challenge of WISH cells with 100 TCID_50_ VSV for 24 h resulted in significant cell lesions. However, pretreatment with 13 subtypes of MiIFN-α attenuated VSV-induced cell lesions, with MiIFN-α2 and MiIFN-α12 demonstrating the strongest effects. Further analysis showed that the effective antiviral concentrations of MiIFN-α2 and MiIFN-α12 in WISH cells were 0.18 × 10 ^4^ IU/mg and 0.3 × 10 ^3^ IU/mg, respectively.

To confirm that the antiviral activity of recombinant MiIFN-α is not limited to VSV, we employed the canine distemper virus (CDV)/Vero system. We selected MiIFN-α2 and MiIFN-α12, because these two subtypes exhibited the strongest antiviral activity against VSV. The results showed that recombinant MiIFN-α2 and MiIFN-α12 significantly inhibited CDV-induced cell lesions. Further analysis showed that 0.145 μg purified MiIFN-α2 protein and 0.28 μg purified MiIFN-α12 could suppress 100 TCID_50_ CDV-mediated cell lesions. These data indicate that MiIFN-α2 exhibits the strongest antiviral activity against VSV and CDV.

## Discussion

The fur animal industry is expanding in the Northeast and Western parts of China. However, the health of fur animals has been compromised by viral infections, such as those with CDV, Parvovirus, and Aleutian mink virus. IFN was first recognized on the basis of its anti-viral activity and only later shown to act primarily as an immunomodulator. IFN-α has been identified from different species, but the biological activity of MiIFN-α has not been tested until now. To determine whether MiIFNα also exhibits antiviral activity similar to IFNs from other species, we examined the ability of recombinant MiIFN-αs to inhibit the replication of VSV.

Using RT-PCR, we successfully amplified the predicted 564 bp mink IFN-α cDNA. The homologies of 13 MiIFN-α subtypes were 93.6–99.3% and 88.8–98.3% at the nucleotide and amino acid sequence levels, respectively. It is surprising that all the subtypes have the same size, whereas size differences have been reported in other species. In 14 pig IFN-αs, multiple sequence alignment revealed a C-terminal deletion of 8 residues in six IFN-α subtypes (IFN-α1, α2, α3, α7, α10 and α11) [17]. The antiviral activities of intact porcine IFN-α genes are approximately 2–50 times higher than those of the subtypes with C-terminal deletions in WISH cells and 15–55 times higher in PK15 cells. In addition, the size of feline IFN-α subtypes are different; feline IFN-α5 has five additional amino acids inserted at position 139, which are not present in the other four subtypes [18]. More than 10 different subtypes of IFN-α have been reported in mice and 16 or more subtypes have been reported to exist in humans. In mouse, the size of IFN-α subtypes is variable. However, the size of the human IFN subtypes is consistent. In mice injected with plasmids encoding murine (Mu)IFN-α1, muIFN-α4 or muIFN-α9, and subsequently challenged with murine cytomegalovirus (MCMV), muIFN-α1 exerted the greatest antiviral effect. In another study, eight different human cell-derived IFN-α subtypes were tested for their antiviral activities. Human (Hu)IFN-α8 was found to be the most potent, whereas HuIFN-α1 exhibited the least antiviral activity. In the current study, we compared the antiviral effects of the MiIFN-α subtypes. There is no evidence to prove that the strength of antiviral activity is related to the size of the IFN-α subtype. Phylogenetic analysis (Figure 2) further indicated that IFN-α was subdivided into two monophyletic lineages: chicken (avian) and mammalian. The mammalian branch can be divided into carnivores and herbivores. The similarity of mink, domestic ferret, Eurasian badger, giant panda, dog, fox, and cat IFN-αs is consistent with their grouping within the carnivore monophyletic group (which is distinct from other herbivores IFN-αs). Indeed, both gene conversion and gene duplication have shaped the evolution of the IFN-α gene family in eutherian species [19]. The features of MiIFN-α subtype sequences provide more support for this view.

IFN has a broad-spectrum of antiviral effects and represents an ideal choice for the development of antiviral drugs. IFN was the first cytokine approved by the US Food and Drug Administration for clinical application. Among the members of the IFN family, IFN-α has relatively higher antiviral activity. IFN-α has been identified from different species, including birds, rodents, and primates [20]. IFN-α is a multi-gene family, with each member located on the same chromosome within a certain region [19]. One unique characteristic of the IFN-α gene is that it has only one open reading frame (ORF) without any introns. For example, dog has 8 IFN-α genes, cat has 5 IFN-α genes, giant panda has 12 IFN-α genes, and the marmot has 10 IFN-α genes. For each subtype of IFN-α gene of the same species, the sequence of the PCR product is the same whether using genomic DNA or cDNA as the template. In this study, we cloned 13 MiIFN-α genes, including six functional genes and two pseudogenes. In vitro assays showed that only the product of the functional IFN gene had antiviral activity. The reason for the genetic diversity of IFN-α during evolution is still unclear, but different subtypes have distinct biological activities [18]. Tan *et al*. cloned 12 IFN-α genes from the giant panda and found that IFN-α8, IFN-α4, and IFN-α10 had higher activity whereas IFN-α11 had low biological activity in 293 cells (human renal epithelial cells transfected with adenovirus E1A gene) [17]. Furthermore, IFN-α3, IFN-α4, and IFN-α8 demonstrated higher activity in B6 cells, whereas IFN-α3, IFN-α7, and IFN-α10 showed higher activity in K562 cells [18]. Taira *et al*. cloned 5 dog IFN-α genes and found that rCaIFN-α8 demonstrated high anti-VSV activity. In addition, the anti- canine adenovirus (CAV)-1 activity of rCaIFN-α8 in Madin-Darby canine kidney (MDCK) cells was 33–666 times higher than the anti-VSV activity, but rCaIFN-α8 had no effects on CHV-1 [21]. Taken together, these data suggest that IFN-α has many subtypes, most of which have antiviral activity in different types of cells. Although the IFN-α family has many pseudogenes that can be transcribed into mRNA, these pseudogenes have no anti-viral function. The potential roles of the pseudogenes of IFN-α remain to be determined.

Effective induction of IFN expression plays an important role during the immune response to viral infection. IFN-α is a multi-gene family consisting of different subtypes with high homology and similar function. However, recent studies have demonstrated that there are functional differences among the subtypes, probably due to the different amino acid sequences. Indeed, our 13 cloned MiIFN-αs demonstrated a variety of constituent amino acids in each subtype. MiIFN-α4–13 had two N-glycosylation sites, whereas MiIFN-α1–3 had only one N-glycosylation site. IFN-α1–3, IFN-α5, IFN-α7, IFN-α10, IFN-α11, and IFN-α13 had 9 cysteine residues, whereas IFN-α4, IFN-α6, IFN-α8, IFN-α9, and IFN-α12 contained only 8 cysteine residues. Antiviral activity analysis showed that only IFN-α1, IFN-α2, IFN-α3, IFN-α8, IFN-α9, and IFN-α12 had antiviral function. It will be interesting to investigate whether N-glycoslylation sites are essential for the antiviral activity of IFN-α.

## Conclusions

In summary, in this study for the first time, we have cloned 13 subtypes of the MiIFN-α gene and successfully expressed them in *E. coli*. Most of the purified recombinant MiIFN-α subtypes demonstrated antiviral activity against VSV and CDV, and MiIFN-α2 exhibited the highest antiviral activity. Therefore, the MiIFN-α2 subtype is a promising candidate for the development of effective antiviral drugs for the fur animal industry.

## Methods

### Reagents

VSV was provided by Dr. Haidong Zhi from the Institute of Harbin Veterinary Research, Chinese Academy of Agricultural Sciences (CAAS). Canine distemper virus (CDV) strains were from commercial CDV-3 vaccine strains. WISH and Vero cells were purchased from the Chinese Academy of Sciences Shanghai Cell Bank. Plasmid pProEX HTb, *E. coli* JM109/BL21, and TRIzol were purchased Invitrogen (USA). RPMI 1640 medium was purchased from GIBCO. Lymphocyte isolation reagent was from the Chinese Academy of Medical Sciences. ExTaq polymerase, AMV reverse transcriptase, and Concanavalin A (Con A) were purchased from Sigma (USA). The Ni-NTA protein purification system and anti-6xHis monoclonal antibodies were purchased from Invitrogen. PCR primers were synthesized by Shanghai Ying-Jun Biotech.

Six-month-old minks were purchased from a fur animal farm in the Jilin Province of China and housed in boxes at the animal house of the Central Laboratory for Animal Diseases of the Institute of Special Animal and Plant Sciences. All animal work and experimental procedures were performed according to the regulations for the administration of affairs concerning experiental animals, which was approved by the state council on October 21, 1988 and promulgated by decree No. 2 of the State Science and Technology Commission on November 14, 1988.

### RT-PCR and analysis of IFN-α sequences

Peripheral blood lymphocytes (PBMCs) were separated under sterile conditions from healthy minks, stimulated with 25 μg/mL ConA for 24 h, centrifuged, and resuspended in 1 mL TRIzol reagent. Total RNA was isolated by the phenol/chloroform method as described previously [22,23]. cDNA was synthesized from total RNA using Oligod (T)_15_ primer, and PCR was performed with cDNA and primers to amplify IFN-α cDNA [16]. The primer sequences are shown in Table 3. The PCR products were subcloned into the pGEM-T vector for sequencing at Shanghai Ying-Jun BioTech (Invitrogen). The amino acid sequences of the MiIFN-αs ORF were aligned with that of IFN-αs of arctic fox, raccoon, and dog IFN-α from GenBank using DNAStar 5.0 software. A multi-species phylogenetic tree based on the nucleotide sequences of the various IFNs was constructed with DNAStar 5.0 MegAlign software. Signal peptides were predicted using online SignalP 4.1 server (http://www.cbs.dtu.dk/Services/SignalP/). The glycosylation sites were predicted using the online 1.0 NetNGlyc Server.

**Table 3:** **Primers used in MiIFN-α and mature peptide (mMiIFN-α) gene PCR assays**

**Gene fragment**	**Predicted size (bp)**	**Primers**	**Tm(** **°** **C)**
MiIFN-α1–13	564	P1 5′-ATGGCCCTGCCCTGCTCCT- 3′	50.1
		P2 5′-TCACTTCCTGCTCCGCAATC-3′	
mMiIFN-α1–13	495	P3 5′- CGGGATCCTGTGACCTGCCTCAG-3′	55
		P4 5′- CCAAGCTTTCACTTCCTGCTCCGCAAT-3′	

### Construction of mMiIFN-α1–13 expression vector

The PCR products were separated by 1.5% agarose gel electrophoresis. The gel purified DNA was cut with *Bam*H I and *Hin*d III, ligated into the pProEX HTb vector, and transformed into competent *E. coli* /BL 21 cells. Positive clones were validated by PCR and restriction enzymes digestion and sequenced by Shanghai Ying-Jun BioTech. The recombinant plasmid was named pHTb/MiIFN-α.

### Expression and purification of recombinant mMiIFN-α1–13

pHTb/MiIFN-αs were transformed into *E. coli* BL21 competent cells and selected with ampicillin. Positive clones were inoculated in 5 ml liquid Luria broth (LB) media and cultured at 37°C. When the optical density at 600 nm (OD_600_) reached 0.5, 1 mmol/L IPTG was added to induce protein expression. For purification, the bacteria were centrifuged at 10,000 rpm for 10 min. Following several cycles of freezing and thawing, the bacterial pellet was mixed with 1 mg/mL lysozme and lysed by sonication on ice. After collection by centrifuging at 4°C at 10,000 rpm for 10 min, the pellet was washed with buffer (1% Triton X-100, 50 mmol/ml Tris · HCl pH 8.0, 100 mmol/mL NaCl) and dissolved in 8 mol/L urea buffer. Then 50% NI –NTA (4:1) was added and incubated on ice with slow mixing using a magnetic stirrer blender. Finally, the mixture was loaded onto a chromatography column (Novagen) and eluted by serial washing (using pH 8.0, pH 6.3, and pH 4.5 50 Mm Tris · HCl buffer containing 8 M urea). The eluents were separated by 12% SDS-PAGE and transferred onto a PVDF membrane. The membrane was blocked with 5% milk in TBST (0.1 phosphate buffer contain 0.5% Tween20) and incubated with mouse anti-6xHis monoclonal antibody (1:300) at 4°C overnight, followed by incubation with goat anti-mouse IgG-HRP (1:2000) at room temperature for 1 h. Finally, the membrane was developed using the TMB Chromogenic Reagent (Sigma, USA).

### Antiviral activity assay

The antiviral activity of 0.5 mg/mL recombinant IFN-α was determined by cytopathic inhibition assay. WISH cells were plated in 96-well plates and incubated at 37°C with 5% CO_2_ for 12–18 h. Diluted recombinant IFN-α was added to the cell monolayer and incubated at 37°C with 5% CO_2_ overnight. Next, the cells were challenged with 100 TCID_50_ VSV, and the plates were incubated at 37°C for 1–2 days. VSV-induced cytopathic effects were assessed by microscopic examination, and IFN-α concentrations were expressed as the inverse dilution that provided protection of 50% of the cells from VSV-induced cytopathic effects (CPE_50_).

In addition, the antiviral activity of recombinant IFN-α was determined by the inhibition of CDV propagation in Vero cells. The titer of CDV TCID _50_ was determined by reference for Reed-Muench method. Vero cells were plated in 24-well plates until the formation of a monolayer. Diluted recombinant IFN-α was added to the cell monolayer and incubated at 37°C with 5% CO_2_ overnight. Next, the cells were challenged with 100 TCID_50_ CDV, and the plates were incubated at 37°C for 1–2 days. The CDV-induced cytopathic effects were assessed by microscopic examination, and IFN-α concentrations were expressed as the inverse dilution that provided protection of 50% of the cells from CDV-induced cytopathic effects (CPE_50_).
